# Using logic regression to characterize extreme heat exposures and their health associations: a time-series study of emergency department visits in Atlanta

**DOI:** 10.1186/s12874-021-01278-x

**Published:** 2021-04-26

**Authors:** Shan Jiang, Joshua L. Warren, Noah Scovronick, Shannon E. Moss, Lyndsey A. Darrow, Matthew J. Strickland, Andrew J. Newman, Yong Chen, Stefanie T. Ebelt, Howard H. Chang

**Affiliations:** 1 Department of Biostatistics and Bioinformatics, Emory University, Atlanta, USA; 2 Department of Biostatistics, Yale University, New Haven, USA; 3 Gangarosa Department of Environmental Health, Emory University, Atlanta, USA; 4 School of Community Health Sciences, University of Nevada Reno, Reno, USA; 5 Research Applications Laboratory, National Center for Atmospheric Research, Boulder, USA; 6 Department of Biostatistics, Epidemiology and Informatics, University of Pennsylvania, Philadelphia, USA

## Abstract

**Background:**

Short-term associations between extreme heat events and adverse health outcomes are well-established in epidemiologic studies. However, the use of different exposure definitions across studies has limited our understanding of extreme heat characteristics that are most important for specific health outcomes or subpopulations.

**Methods:**

Logic regression is a statistical learning method for constructing decision trees based on Boolean combinations of binary predictors. We describe how logic regression can be utilized as a data-driven approach to identify extreme heat exposure definitions using health outcome data. We evaluated the performance of the proposed algorithm in a simulation study, as well as in a 20-year time-series analysis of extreme heat and emergency department visits for 12 outcomes in the Atlanta metropolitan area.

**Results:**

For the Atlanta case study, our novel application of logic regression identified extreme heat exposure definitions that were associated with several heat-sensitive disease outcomes (e.g., fluid and electrolyte imbalance, renal diseases, ischemic stroke, and hypertension). Exposures were often characterized by extreme apparent minimum temperature or maximum temperature over multiple days. The simulation study also demonstrated that logic regression can successfully identify exposures of different lags and duration structures when statistical power is sufficient.

**Conclusion:**

Logic regression is a useful tool for identifying important characteristics of extreme heat exposures for adverse health outcomes, which may help improve future heat warning systems and response plans.

**Supplementary Information:**

The online version contains supplementary material available at 10.1186/s12874-021-01278-x.

## Introduction

Extreme heat events have significant public health impacts as demonstrated, for example, by historical heat waves in Chicago [1, 2] and Europe [3, 4]. Recent epidemiology studies have also found consistent short-term associations between extreme heat events and various cause-specific mortality [5–9] and morbidity outcomes [10–12]. However, synthesizing existing evidence has been challenging because of the use of various exposure definitions in previous studies [13, 14]. How extreme temperatures become hazardous can vary across locations due to differences in societal and biological adaptations [15], as well as across outcomes due to differences in physiological mechanisms [16]. Identifying extreme heat characteristics that are most important for specific health outcomes or vulnerable subpopulations may help improve heat warning systems and response plans.

Extreme heat events are typically characterized by their exceedance over an intensity *threshold* and their sustained *duration*. For example, previous studies have defined heat waves as a period where temperature exceeds the 98th percentile over two or more consecutive days [17–19]. Many other relative (or absolute) thresholds and durations have been used to define extreme heat in health studies [20]. Furthermore, the choice of *heat metric* represents another source of variation across studies. Daily maximum and average temperatures have been the most commonly used heat metrics. But there is increasing interest in investigating apparent temperature or wet-bulb temperature [21] that may better reflect human discomfort, and minimum temperature that reflects night-time exposure [22–24].

The current approach of assessing effect heterogeneity due to exposure definitions within a study involves examining different heat metrics, different extreme thresholds, and different durations one-at-a-time. Studies often also need to take into account statistical power in exposure definition due to the low frequency of extreme heat days [25]. There has been limited work in leveraging health data directly to develop exposure definitions for extreme heat. Recently, machine learning methods have been applied to predict adverse health outcomes using meteorological variables [26]. However, the resulting algorithms can be difficult to interpret in terms of the two key characteristics of duration and intensity threshold. These approaches may also suffer from the lack of rigorous control for confounders, making results more difficult to translate into causal effects for subsequent intervention and impact analysis.

In this paper, we examine the use of *logic regression* [27–29], a machine learning method, to help identify characteristics of extreme heat events that are associated with adverse health outcomes. Logic regression estimates a decision tree constructed using Boolean combinations of binary predictors. Logic regression has been utilized extensively in genetic association studies for identifying high-dimensional interactions [30, 31], and has recently been extended to other exposures [32–34]. We show how logic regression provides a data-driven approach to construct a daily *extreme heat exposure indicator* that is binary (i.e., presence versus absence of the exposure) and can capture impacts of *sustained* extreme exposure over several days (i.e., heat waves). We evaluated the performance of the approach in simulation studies and applied the method to a 20-year time-series analysis of daily emergency department (ED) visits in Atlanta, Georgia.

## Materials and methods

### Atlanta emergency department visit and meteorology data, 1993–2012

Patient-level ED visits data were obtained directly from hospitals within the 20-county Atlanta metropolitan area from 1993 to 2004 and then from the Georgia Hospital Association from 2005 to 2012. For some outcomes, secondary diagnoses were also included because they showed stronger associations with temperature in a previous Atlanta analysis [35]. The selected health outcomes are internal causes (INTERN), heat illness (HEAT), ischemic stroke (STK), fluid and electrolyte imbalances (FLEL), all renal disease (RENAL), acute renal failure (ARF), all circulatory system disease (CIRC), hypertension (HT), myocardial infarction (MI), congestive heart failure (CHF), ischemic heart disease (IHD), and diabetes (DIA). Table 1 provides summary statistics of daily ED visits and ICD-9 codes for each outcome. Only admissions during the warm seasons (May 1st to September 30th) were used in this analysis because of our interest in comparing extreme heat events versus non-event warm days.

**Table 1:** Descriptive statistics for daily emergency department visits during May to September in the 20-county Atlanta Metropolitan area, 1993–2012. Outcomes are ordered by decreasing total counts

Disease	ICD-9 Code(s)	Mean Daily ED Visits	Standard Deviation	Min	Max
All Internal Causes	001–799	2553	1328	192	5557
Diabetes	249, 250	215	171	0	630
Fluid and Electrolyte Imbalance^a^	276	162	116	0	413
All Circulatory System Diseases	390–459	623	467	3	1652
Hypertension	401–405	491	408	0	1407
Ischemic Heart Disease	410–414	120	87	0	327
Myocardial Infarction^a^	410	14	8	0	40
Congestive Heart Failure	428	74	58	0	245
Ischemic Stroke	433–437	23	15	0	67
All Renal Diseases	580–593	139	118	0	447
Acute Renal Failure^a^	584	36	42	0	155
Heat-related Illnesses^a^	992.5	4	6	0	64

^a^Diagnosis based on primary ICD code only

Hourly ambient air (dry-bulb) temperature, dew-point temperature, and apparent temperature were obtained at the Atlanta Hartsfield International Airport weather station from the National Climatic Data Center from 1993 to 2012. We used airport monitor due to its high-quality, complete temporal observations and central location in the study area, which has little variation in elevation. Apparent temperature in °C was defined as − 1.3 + 0.92 T + 2.2e, where T is ambient air temperature (°C) and e is water vapor pressure (kPa) [36]. We considered six heat metrics: daily maximum (MX), minimum (MN), and average (Avg) of either dry-bulb temperature or apparent temperature (AT). For each daily temperature variable, we created binary *extreme indicators* at the 95th, the 98th or the 99th percentile thresholds based on observations over the 20-year study period. Specifically, the extreme indicator takes the value 1 when the temperature value exceeds the percentile threshold.

### Time-series model for ED visits and temperature

We first describe the quasi-Poisson log-linear model used to estimate short-term associations between daily ED visit counts and extreme temperature [36]. Following our previous analysis of heat waves and ED visits in Atlanta, [37], denote ${\mu}_t^a$ the mean ED visit count for adverse health outcome *a* on day *t*. The time-series model is given by

$$ \ln \left({\mu}_t^a\right)={\beta}_0+\beta H\left({\boldsymbol{X}}_t\right)+ ns\left({T}_t\right)+ ns\left({\overline{T}}_t\right)+ ns\left( DP{T}_t\right)+{\alpha}_{0, yea{r}_t}+f\left( DAT{E}_t\right)\times {\alpha}_{1, yea{r}_t}+\sum \limits_{i=1}^6{\lambda}_i DO{W}_{ti}+\sum \limits_{j=1}^2{\delta}_j HOLIDA{Y}_{tj}+\sum \limits_{k=1}^{32}{\gamma}_k HO\mathrm{S} PITA{L}_{tk}. $$

Our parameter of interest *β* is the log relative risk (RR) associated with a binary extreme heat exposure *H*(***X***_*t*_). The use of logic regression to define *H*(***X***_*t*_) is described in detail later in Section 2.3. The above time-series model adjusts for *non-extreme* continuous same-day temperature (*T*_*t*_), average temperature over the last 3 days (lag 1, lag 2 and lag 3) (${\overline{T}}_t$), and same-day maximum dew-point temperature (*DPT*_*t*_) to reflect the discomfort level due to the humidity. Specifically, in primary analyses, we defined the continuous temperature *T*_*t*_ and ${\overline{T}}_t$ by *truncating* the value at the extreme heat threshold (the 95th, 98th, or 99th percentile), i.e., setting any daily temperature value above the threshold to be the threshold value. In a sensitivity analysis, we also examined the use of the entire range of observed (i.e., non-truncated) temperature; in this case, *H*(***X***_*t*_) can be interpreted as the “added” impact of extreme heat beyond the continuous temperature effect. The use of truncated continuous exposure is to provide better interpretation of *β* because *H*(***X***_*t*_) only reflects the temperature effect beyond the threshold. Moreover, the tail of exposure-response function is often difficult to estimate due to sparse data and can be more sensitivity to influential observations. Hence, adjusting for non-truncated temperature may result in *βH*(***X***_*t*_) accommodating for mis-specification of the exposure-response functions *ns*(*T*_*t*_) and $ns\left({\overline{T}}_t\right)$ at the extreme tail. When apparent temperature is the exposure of interest, we did not include dew-point temperature in the model. To model the possible non-linear effect of meteorology, natural cubic splines, denoted by *ns (.),* were used for ${T}_t,{\overline{T}}_t,$ and *DPT*_*t*_ with 2 equidistant internal knots.

We adjusted for long-term temporal trend in the time-series model as follows. Within a year, seasonal variation (from May to September), denoted by *f*(*DATE*_*t*_), was modeled smoothly with natural cubic splines and monthly knots. We included year-specific indicators ${\alpha}_{1, yea{r}_t}$ and their interactions with the seasonal pattern in our model to allow for between-year variation. We also adjusted for day of week effect using indicators *DOW*_*ti*_ and for federal or state holiday using indicators *HOLIDAY*_*tk*_. Finally, *HOSPITAL*_*tk*_ represents indicator variables for whether hospital *k* contributes to the ED visits counts on day *t;* these indicators were used to account for potential temporal drops in ED counts due to missing data from individual hospitals.

### Logic regression

Logic regression is an adaptive regression method that attempts to construct predictors as Boolean combinations of binary covariates. It constructs a simple decision tree or a set of decision trees (multiple trees) with binary predictors connected by *and* (∧), *or* (⋁), and *not* (^c^) operators. For example, consider the following four binary extreme heat indicators based on minimum apparent temperature (ATMN),

$$ {X}_1=\mathrm{lag}0\left({\mathrm{today}}^{\prime}\mathrm{s}\right)\kern0.28em \mathrm{ATMN}>98\mathrm{thpercentile}, $$

$$ {X}_2=\mathrm{lag}1\left({\mathrm{yesterday}}^{\prime}\mathrm{s}\right)\kern0.28em \mathrm{ATMN}>98\mathrm{thpercentile}, $$

$$ {X}_3=\mathrm{lag}2\kern0.28em \mathrm{ATMN}>98\mathrm{thpercentile}, $$

and

$$ {X}_4=\mathrm{lag}3\kern0.28em \mathrm{ATMN}>98\mathrm{thpercentile}, $$

where we suppress the subscript *t* for presentation purposes. A simple extreme heat definition may be *H*(***X***_*t*_) = *X*_1_ ∧ *X*_2_ ∧ *X*_3_, which describes a period of 3-consecutive days with high temperature. Hence, using these set of indicators, we can potentially capture both lagged and sustained effects of extreme heat. In our application, we focus on the use of the single tree model to estimate *H*(***X***_*t*_). In another example, $H\left({\boldsymbol{X}}_t\right)={X}_1^c\wedge {X}_2\wedge {X}_3$ describes a period of 2-consecutive days with high temperature, excluding the lag-0 day. This is different from *X*_2_ ∧ *X*_3,_ which does not place a restriction on whether X_1_ being 0 or 1. We also note that *H*(***X***_*t*_) is a binary exposure variable and offers better interpretation for risk associations compared to a more naïve approach of including *X*_1_, *X*_2_, *X*_3,_ and X_4_, as well as their interactions, jointly in a model.

Different logic trees may result in the same classification of days. For example, the two trees identified by logic regressions:

$$ {H}_1\left({\boldsymbol{X}}_t\right)=\left({X}_1^c\wedge {X}_2\wedge {X}_3\right)\vee \left({X}_2\wedge {X}_3\wedge {X}_4\right)\mathrm{and}{H}_2\left({\boldsymbol{X}}_t\right)=\left({X}_1^c\vee {X}_4\right)\wedge \left({X}_2\wedge {X}_3\right). $$

give the same classification according to the distributive law (i.e., *A* ∧ (*B* ⋁ *C*) = (*A* ∧ *B*) ⋁ (*A* ∧ *C*)). To aid in interpretation of logic regression results, we used the following scheme to describe *H*(***X***_*t*_). The Boolean combinations of heat indicators generated by logic regression can always be expressed by a series of logic statements joined by the ⋁ (or) operators. Hence in the above example, *H*_1_(***X***_*t*_) =(X_1_^c^ ∧ X_2_ ∧ X_3_) ⋁ (X_2_ ∧ X_3_∧ X_4_) is the preferred form with logic statements ${X}_1^c\wedge {X}_2\wedge {X}_3$ and *X*_2_ ∧ *X*_3_ ∧ *X*_4_.

The ‘*Logicreg’* package in R was used to fit logic regression models. Estimation was based on simulated annealing as the optimization algorithm to stochastically explore all ${2}^{2^k}$ Boolean combinations for k predictors. Ten-fold cross validation was used to select the size of the tree (i.e., number of leaves) and reduce issues related to overfitting.

### A multi-stage estimation approach

We applied logic regression to time-series analysis of ED visit data in a three-stage approach. In the first stage, for each temperature variable (i.e., ambient air temperature and apparent temperature) and metric (i.e., maximum, minimum, or average), a quasi-Poisson log-linear model *without H*(***X***_*t*_) was fit.

In the second stage, the Pearson residuals from the first-stage model were used to identify the Boolean combination of different extreme heat indicators at various lags. The Pearson residuals were calculated as

$$ {r}_t^a=\frac{Y_t^a-{\hat{\mu}}_t^a}{\sqrt{V\left({\hat{\mu}}_t^a\right)}} $$

where ${Y}_t^a$ and ${\hat{\mu}}_t^a$ are, respectively, the observed number of daily ED visits and the predicted mean number of daily ED visits for outcome of interest *a* on day *t*, and $V\left({\hat{\mu}}_t^a\right)$ is the product of ${\hat{\mu}}_t^a$ and the dispersion parameter. In the first stage we removed effects of continuous same-day and lagged temperatures, as well as other temporal trends. Because Pearson residual represents a scaled difference between the observed and expected counts, the logic regression tree *H*(***X***_*t*_) estimated using Pearson residuals aims to captures additional lagged and sustained associations due to extreme temperature not explained by the base model. Finally, in the third stage, we refit the full time-series model with *H*(***X***_*t*_) and all other covariates.

## Simulation study

### Simulation setup

We performed a simulation study to assess the performance of logic regression a our multi-stage procedure in detecting the structure of extreme heat exposures and in estimating associations with health outcomes. Let X_1_, X_2_, and X_3_ be binary indicators for the minimum apparent temperature exceeding the 98th percentile threshold on lag 0, lag 1, and lag 2 day, respectively. We considered three different true *H*(***X***_*t*_) exposures:

- E1 Same-day effect: *H*(***X***_*t*_) = *X*_1_,
- E2 Sustained 2-day effect: *H*(***X***_*t*_) = *X*_1_ ∧ *X*_2_, and
- E3 Sustained 2-day lagged-only effect: $H\left({\boldsymbol{X}}_t\right)={X}_1^c\wedge {X}_2\wedge {X}_3$.

We considered three different health outcomes with different sample sizes, temporal patterns, and overdispersion (CIRC, RENAL, and HEAT). For each disease, we first fit the time-series model with all confounders as described in Section 2.1 with the Atlanta ED and meteorology data during 1993–2012 to obtain the baseline mean daily ED visits. We assumed a true relative risk (RR) of 1.01 or 1.05 for *H*(***X***_*t*_) and simulated daily ED visit counts from a negative-binomial distribution and observed meteorology data using the time-series model given in Section 2.2. Regression coefficients and overdispersion were based on estimated values from models fitted with real data. We then applied the three-stage algorithm described in Section 2.4 to the simulated data and estimated the log RR of interest.

We ran the simulation 100 times for each scenario. The relative bias and relative root mean squared error (RRMSE) were used to examine the performance of the proposed approach. Relative bias and RRMSE were calculated as

$$ Rel\mathrm{a} tive\ bias=\frac{\frac{1}{100}{\sum}_{i=1}^{100}\left(\hat{R{R}_i}-R{R}_{true}\right)}{R{R}_{true}}, $$

$$ RRMSE=\frac{\sqrt{\frac{1}{100}{\sum}_{i=1}^{100}{\left(\hat{R{R}_i}-R{R}_{true}\right)}^2}}{R{R}_{true}}. $$

Here, ${\hat{RR}}_i$ is the estimated RR of $\hat{H}\left({\boldsymbol{X}}_t\right)$ based on logic regression from the i^th^ simulation and *RR*_*true*_ is the true RR for each scenario. We also estimated the sensitivity and specificity by comparing days indicated by $\hat{H}\left({\boldsymbol{X}}_t\right)$ to be exposed/unexposed to the true exposure status given by E1, E2, or E3.

### Simulation study results

Results from the simulation study are summarized in Table 2. We found that performance of the proposed method was better with larger RRs and for outcomes with larger daily event counts (e.g., comparing all renal diseases versus heat-related illnesses). We also ran the same simulation using the non-truncated continuous temperature and found similar results. Among the three different exposure scenarios, logic regression performed best for scenario E1 (single-lag, same-day) and worst in E3 (sustained two-day lagged consecutive exposure). The frequency of the extreme heat event may explain the different performances; the frequency of occurrence in our Atlanta study for E1, E2, and E3 were 146, 73, and 29 days, respectively. Importantly, we also found that in our Atlanta ED visits application, the average bias was negative, indicating that the effect estimate was attenuated towards the null. This is likely due the presence of exposure misclassification: when sensitivity/specificity is not 100%, some days are classified incorrectly as exposed/unexposed.

**Table 2:** Summarized simulation study results of the performance of logic regression

Disease	True Relative Risk	Exposure Scenario	Sensitivity^a^	Specificity^a^	Relative bias		Relative root mean square error (RRMSE)	
					Logic regression	Known *H*(***X***_*t*_)	Logic regression	Known *H*(***X***_*t*_)
All Circulatory System Diseases	1.01	E1	50%	99%	−0.56%	−0.01%	1.09%	0.55%
		E2	35%	98%	−0.84%	− 0.04%	1.59%	0.69%
		E3	23%	98%	−1.13%	−0.28%	2.02%	0.85%
	1.05	E1	100%	100%	−0.11%	− 0.11%	0.69%	0.69%
		E2	99%	100%	−0.03%	0.03%	0.99%	0.71%
		E3	98%	100%	−0.09%	−0.02%	1.23%	1.00%
All Renal Diseases	1.01	E1	34%	99%	−0.40%	0.36%	1.39%	1.10%
		E2	36%	98%	−0.27%	0.10%	2.03%	1.26%
		E3	26%	98%	−0.92%	−0.22%	3.03%	1.61%
	1.05	E1	93%	100%	−0.14%	0.12%	1.75%	1.21%
		E2	82%	100%	−1.00%	0.18%	3.29%	1.16%
		E3	73%	99%	−1.37%	−0.16%	3.31%	1.57%
Heat-Related Illnesses	1.01	E1	19%	99%	−0.57%	0.35%	6.58%	5.61%
		E2	29%	98%	0.30%	1.05%	12.02%	6.93%
		E3	17%	98%	0.09%	0.91%	16.21%	9.03%
	1.05	E1	33%	98%	−2.53%	−0.23%	8.43%	6.44%
		E2	30%	98%	−0.83%	0.45%	11.66%	6.40%
		E3	23%	98%	−3.07%	−0.92%	16.42%	9.10%

^a^Sensitivity is defined as the proportions of days assigned as exposed using the exposure metric estimated from logic regression among days assigned as exposed using the true exposure metric (E1, E2, or E3) for simulating health data. Specificity is defined similarly for days assigned as unexposed. For each simulation scenario, the sensitivities and specificities reported are averaged across 100 simulations

## Application to the Atlanta ED visit data

### Primary analyses

We applied the multi-stage algorithm described in Section 2.4 separately for each heat metric: MX, MN, and Avg of temperature (T) or apparent temperature (AT), and separately for each threshold (95th, 98th, 99th percentile). Extreme binary indicators were also defined for up to three lagged days. The model with the smallest quasi-AIC was selected.

Table 3 summarizes the structure of the extreme heat exposures *H*(***X***_*t*_) and their associations with ED visits. Supplementary Figure 1 shows the structure of the logic regression tree *H*(***X***_*t*_) for selected outcomes. Logic trees can be read “bottom-up” to construct a corresponding logic statements where the tree split is given by the Boolean statement and/or. Given a logic regression tree, individual days in our 20-year study period data were divided into two groups and we defined the reference (i.e., *H*(***X***_*t*_) = 0) as the type of days more frequently observed.

**Table 3:** Summary of extreme heat exposure estimated by logic regression and their short-term associations with warm-season emergency department visits in Atlanta, 1993 to 2012. Relative risk estimates and 95% confidence intervals (CI) were from time-series models adjusting for truncated continuous temperature. Within each outcome, each row of the extreme heat exposure corresponds to an “or” statement derived from the logic regression tree. “Not” statement is indicated by a superscript *c*

ED Visit Outcome	Heat Metric and Quantile	Extreme Heat Exposure	lag0	lag1	lag2	lag3	Frequency (days)	Relative Risk (95% CI)
HEAT	ATMN95	(lag0 and lag1)	Y	Y			237	**1.400 (1.269, 1.544)**
		OR (lag1 and lag3)		Y		Y		
FLEL	ATMN95	lag1		Y			335	**1.030 (1.016, 1.045)**
RENAL	ATMN95	(lag0 and lag3)	Y			Y	255	**1.025 (1.011, 1.039)**
		OR (lag2^C^ and lag3)			N	Y		
ARF	ATMN95	lag0 and lag1	Y	Y			190	**1.052 (1.021, 1.085)**
STK	TMX98	lag0 and lag1 and lag2^C^ and lag3	Y	Y	N	Y	5	**1.257 (1.061, 1.477)**
CIRC	ATMX95	lag1		Y			363	0.996 (0.987, 1.004)
HT	ATMX95	lag0 and lag1 and lag3^C^	Y	Y		N	128	**0.988 (0.977, 0.999)**
		OR (lag1 and lag2 and lag3^C^)		Y	Y	N		
IHD	TMN99	lag0	Y				63	1.013 (0.986, 1.041)
MI	TMN99	lag0	Y				63	1.044 (0.964, 1.131)
CHF	ATMN98	lag0	Y				146	0.976 (0.947, 1.007)
DIA	TMN95	lag2			Y		238	1.009 (0.996, 1.021)
INTERN	ATMN95	lag3				Y	335	**1.008 (1.002, 1.015)**

*HEAT* heat illness, *STK* ischemic stroke, *ARF* Acute renal failure, *RENAL* all renal disease, *FLEL* Fluid and electrolyte imbalance, *CIRC* all circulatory system disease, *CHF* Congestive heart failure, *IHD* Ischemic heart disease, *MI* Myocardial infarction, *DIA* Diabetes, *HT* Hypertension, *ITERN* all internal causes)

Overall, we found several positive associations with exposures. For example, for heat-related diseases (HEAT), extreme heat exposure was defined as days where (1) lag 0 and lag 1 ATMIN are above the 95% percentile or (2) lag 1 and lag 3 ATMIN is above the 95% percentile. This exposure was associated with an increase in mean HEAT ED visits by 40% (95% CI: 27–54%). For acute renal failure (ARF) ED visits, the exposure identified (i.e., recent two days’ ATMIN is above the 95% percentile) was associated with an increase of 5% (95% CI: 2–9%). For all renal diseases, the exposures identified were more complicated and was associated with an increase of ED visits by 2% (95% CI: 1–4%). Associations with circulatory disease were generally null, except for ischemic stroke and hypertension, for which we found a negative association with extreme heat exposure

### Sensitivity analyses

We also conducted three additional sensitivity analyses. First, we examined the more conventional heat wave definition where duration is defined as at least two consecutive days exceeding the threshold. Result for HEAT, STK, and RENAL outcomes are given in Supplementary Table S1. We found that the conventional heat wave definitions also indicated positive associations, but the magnitude can be attenuated. Second, because logic regression is a machine learning algorithm that optimizes predictability, the lag structure may contain holes (e.g., exceeding the temperature threshold on lag-1 and lag-3, but not lag-2). Based on the exposure lags identified by logic regression as a guide, we defined alternative exposure metrics by filling in gaps and removing the “not” statement. These alternative metrics are defined over consecutive days and may be more interpretable. Results are given in Table S2. For HEAT and RENAL, we found that using alternate exposures that are more extreme (less frequent) continues to estimate positive associations. However, in the case of STK, where the alternate exposures are less extreme, the associations with ED visits were positive, but confidence intervals included the null.

In a second analysis, we replaced the truncated same-day and 3-day moving-average of temperature heat metric with the original variable without truncation. Here the application of logic regression attempts to identify *additional risks* beyond that conferred by the continuous exposure-response function. Results are given in Supplementary Table S2. In general, we found that logic regression identified extreme heat exposure based on similar heat metrics but RR with smaller magnitude. However, the use of truncated continuous temperature, as in our main analysis, was generally associated with better model fit and stronger associations with ED visits. This may be attributed to data sparsity in the extreme tail of the temperature distribution such that when using non-truncated temperature, the tail of the continuous exposure-response function may be mis-specified and has high uncertainty.

## Discussion

In this paper, we propose the use of logic regression to help identify characteristics of extreme heat exposure that are associated with short-term adverse health risks. Our 20-year time-series analysis shows that ED visits for various disease outcomes were associated with exposure identified by logic regression using a multi-stage algorithm. Our motivating hypothesis is that most harmful characteristics of heat exposures are likely to vary between outcomes because of the different vulnerable subpopulations and different pathophysiological mechanisms they may impact. While the strength of association of different exposure definitions varied by outcome, the most health-relevant exposures were generally those characterized by temperatures exceeding a threshold over multiple lags. We also found evidence that apparent temperature and daily minimum temperature gave better model fit, providing support that humidity and night-time exposure are important considerations for quantifying adverse health effects of extreme heat events. However, Armstrong et al. [38] found that adding relative humidity or dewpoint temperature to a model with temperature does not improve model fit.

Though studies on heat waves and cause-specific ED visits are limited compared to cause-specific mortality, our results are consistent with much of the previous research that utilized different definitions. Petitti et al. [39] used three pre-specified temperature trigger points (minimum risk temperature, increasing risk temperature, and excess risk temperature) in Maricopa County Arizona. This study found significant associations with all three trigger points and ED visits for heat related diagnosis but found no association with CVD related outcomes and total ED visits. Another study that looked at high ambient temperature and ED visits in California found that same day ambient temperature was positively associated with heat illness and ARF, but was negatively associated with hypertension [40], as were our results.

For hypertension, we found that the exposures estimated from logic regression tended to be negatively associated with ED visits. Previous studies on heat waves and morbidity outcomes have found similar results. Lim et al. [9] and Sherbakov et al. [41] found that hospital admissions due to hypertension and other cardiovascular related outcomes decreased with increased temperatures. Similarly, a study by Michelozzi et al. [42] found that cardiovascular related morbidity was reduced with higher temperatures, but cardiovascular mortality increased. A possible mechanism for these findings is that blood pressure can decrease from vasodilation and sweating in the summer, thus potentially reducing hypertension related hospital admissions [41, 43].

Our extreme heat exposures did not always identify consecutive days of high temperature as being the most harmful. For example, the *not* logic statement and *missing* lags were selected for several disease outcomes (Table 3 and S2). This may be due to the lack of statistical power as periods with consecutive days of high temperature were less frequent. It is also possible that the risk of consecutive days of high temperature is less harmful compared to periods with more *variable* but high temperature due to awareness of extreme heat events.

We found that different specifications of the base model (i.e., adjusting for truncated versus non-untruncated continuous temperature) can have an impact on the extreme heat exposure identified and its estimated risk ratio. While both types of analyses are common in the literature, associations of extreme heat events from these two approaches should be interpreted differently (i.e., total risks above a certain threshold versus risks in addition to the continuous exposure-response function). This motivated our multi-stage estimation approach such that the base model is specified a priori and the data-driven logic regression is only utilized to explore potential excess risks not explained by the base model. We note that when performing risk assessment of high temperature, both extreme temperature events and the tail of the exposure-response function should be considered.

The main advantage of logic regression is its supervised learning approach for deriving study-specific and outcome-specific exposure definitions that are flexibly constructed by indicators of different extreme heat characteristics. Study population, geographical region, and the set of exposures being considered may contribute to the observed heterogeneity in extreme heat health effects within and across studies. The algorithm also has a user-friendly software package that can efficiently evaluate a large suite of possible exposure definitions to identify those that are most important for individual health outcomes. Compared to regression tree methods, logic regression has two important advantages: (1) the ability to incorporate “and”, “or” and “not” statement between predictors, and (2) the focus on binary classification of a continuous outcome. In contrast, Classification And Regression Trees (CART) will give multiple terminal nodes and the resulting heat metric will likely be very complex because only “and” statement is allowed as the decision tree is split.

Finally, logic regression has several limitations that warrant further investigations. First, during estimation, logic regression can become trapped in a local minimum when many binary predictors are being considered. Hence, model fitting requires a comprehensive evaluation of tuning parameters (e.g., starting temperature, finishing temperature, and cooling schemes) and initial values, which increases computational burden. In our application, the number of binary predictors is considerably smaller than the typical genetic association studies and we were able to evaluate different control parameters for simulated annealing. Second, we only utilized logic regression to select the exposure lag structure given a heat metric and threshold. We found that the current sample size cannot accommodate including all possible heat exposure indicators that are highly correlated. One future direction is to consider additional penalization within logic regression. Third, our multi-stage estimation approach, while allowing us to work with established time-series analysis methodology, does not account for estimation uncertainty associated with the extreme heat metric, which may be important for quantifying associations for rare exposure events. Statistical inference such as pseudolikelihood [44, 45] could be integrated to the current method to account for the ignored uncertainty. Moreover, recent advances in fitting logic regression under a Bayesian framework [46] allows for direct quantification of uncertainties via posterior samples. How to incorporate these uncertainties in a multi-stage health analysis, similar to an exposure measurement error framework, warrants further investigations.

## Supplementary Information

**Additional file 1: Figure S1.** Structure of logic regression tree of extreme heat exposures for selected warm-season ED visit outcomes in Atlanta, Georgia, 1993–2012. **Table S1.** Summary of alternative extreme temperature metrics and their short-term associations with warm-season emergency department visits in Atlanta, 1993 to 2012. **Table S2.** Summary of alternative extreme temperature metrics with consecutive lags and their short-term associations with warm-season emergency department visits in Atlanta, 1993 to 2012. **Table S3.** Summary of extreme heat metrics from truncated continuous versus continuous temperature metric and their short-term associations with warm-season emergency department visits in Atlanta, 1993 to 2012.

## Data Availability

The daily emergency department data used in this study contain protected health information and cannot be shared due to existing data use agreements. Example R code for carrying out the simulation study and analysis are available from the corresponding author upon request.

## Abbreviations

ARF: Acute renal failure
AT: Apparent temperature
CHF: Congestive heart failure
CIRC: Circulatory system diseases
ED: Emergency department
DIA: Diabetes
FLEL: Fluid and electrolyte imbalances
HEAT: Heat illness
HT: Hypertension
IHD: Ischemic heart disease
INTERN: Internal causes
MI: Myocardial infarction
MN: Minimum
MX: Maximum
RENAL: Renal diseases
RR: Relative risk
STK: Ischemic stroke
T: Temperature
