# Supplementary material for: Using logic regression to characterize extreme heat exposures and their health associations: a time-series study of emergency department visits in Atlanta
Source: BMC Med Res Methodol. 2021 Apr 26;21:87. doi: 10.1186/s12874-021-01278-x (PMC8077733; doi:10.1186/s12874-021-01278-x)
Supplement: Supplementary file 1 — Additional file 1: Figure S1. Structure of logic regression tree of extreme heat exposures for selected warm-season ED visit outcomes in Atlanta, Georgia, 1993–2012. Table S1. Summary of alternative extreme temperature metrics and their short-term associations with warm-season emergency department visits in Atlanta, 1993 to 2012. Table S2. Summary of alternative extreme temperature metrics with consecutive lags and their short-term associations with warm-season emergency department visits in Atlanta, 1993 to 2012. Table S3. Summary of extreme heat metrics from truncated continuous versus continuous temperature metric and their short-term associations with warm-season emergency department visits in Atlanta, 1993 to 2012. [file 12874_2021_1278_MOESM1_ESM.docx]

**Supplementary Materials for**

**Using logic regression to characterize extreme heat exposures and their health associations: a time-series study of emergency department visits in Atlanta**

Shan Jiang,^1^ Joshua L Warren,^2^ Noah Scovronick,^3^ Shannon Moss,^1^ Lyndsey A Darrow,^4^ Matthew J Strickland,^4^ Andrew J Newman,^5^ Yong Chen,^6^ Stefanie T Ebelt,^3^ Howard H Chang* ^1^

^1^Department of Biostatistics and Bioinformatics, Emory University

^2^Department of Biostatistics, Yale University

^3^Gangarosa Department of Environmental Health, Emory University

^4^School of Community Health Sciences, University of Nevada, Reno

^5^Research Applications Laboratory, National Center for Atmospheric Research

^6^Biostatistics at the Department of Biostatistics, Epidemiology and Informatics, University of Pennsylvania

*Corresponding author: [howard.chang@emory.edu](mailto:howard.chang@emory.edu)

**Figure S1.** Structure of logic regression tree of extreme heat exposures for selected warm-season ED visit outcomes in Atlanta, Georgia, 1993–2012. The statistics in the top left corner gives the difference in mean Pearson residual predicted by the logic tree. The label of each node represents the indicator of whether a heat metric (e.g., maximum apparent temperature, ATMX) exceeds a threshold (e.g., 95^th^ percentile) on a specific lag day. Black boxes denote the complement of the indicator.


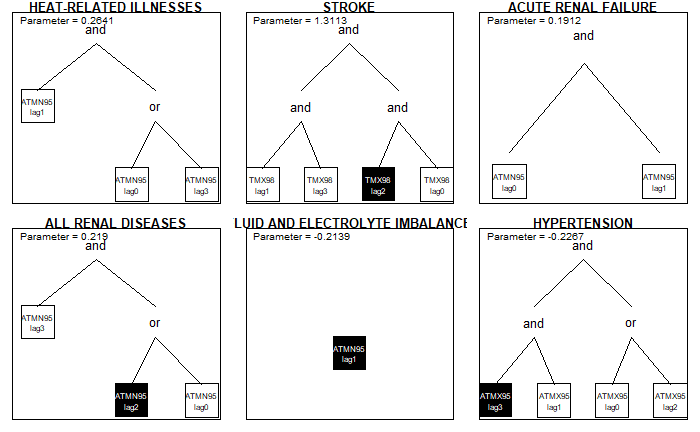


**Table S1.** Summary of alternative extreme temperature metrics and their short-term associations with warm-season emergency department visits in Atlanta, 1993 to 2012. Relative risk estimates and 95% confidence intervals (CI) were from time-series models adjusting for truncated continuous temperature. The exposure identified by logic regression is highlighted blue.

| Disease | Temperature  and  Quantile | Extreme Heat Metric | Relative risk  (95% CI) | Frequency  (days) | Q-AIC | lag0 | lag1 | lag2 | lag3 |
| --- | --- | --- | --- | --- | --- | --- | --- | --- | --- |
| HEAT | ATMN95 | lag0 and lag1 | 1.400 (1.269, 1.544) | 237 | 5919 | Y | Y |  |  |
|  |  | lag1 and lag3 |  |  |  |  | Y |  | Y |
| HEAT | ATMN95 | lag0 and lag1 | 1.301 (1.177, 1.439) | 190 | 5889 | Y | Y |  |  |
| STROKE | TMX98 | lag0 and lag1 and  (not lag2) and lag3 | 1.257 (1.061, 1.477) | 5 | 16747 | Y | Y | N | Y |
| STROKE | TMX98 | lag0 and lag1 | 1.027 (0.955, 1.103) | 61 | 16717 | Y | Y |  |  |
| RENAL | ATMN95 | lag0 and lag3 | 1.025 (1.011, 1.039) | 255 | 18888 | Y |  |  | Y |
|  |  | (not lag2) and lag3 |  |  |  |  |  | N | Y |
| RENAL | ATMN95 | lag0 and lag1 | 1.033(1.015, 1.050) | 190 | 18886 | Y | Y |  |  |

**Table S2.** Summary of alternative extreme temperature metrics with consecutive lags and their short-term associations with warm-season emergency department visits in Atlanta, 1993 to 2012. Relative risk estimates and 95% confidence intervals (CI) were from time-series models adjusting for truncated continuous temperature. The exposure identified by logic regression is highlighted blue. Alternative exposures are defined by using all lags identified by logic regression.

|  | Disease | Temperature  and  Quantile | Extreme Heat Metric | Relative risk  (95% CI) | Frequency  (days) | Q-AIC | lag0 | lag1 | lag2 | lag3 |
| --- | --- | --- | --- | --- | --- | --- | --- | --- | --- | --- |
| Logic regression | HEAT | ATMN95 | lag0 and lag1 | 1.400 (1.269, 1.544) | 237 | 5919 | Y | Y |  |  |
|  |  |  | lag1 and lag3 |  |  |  |  | Y |  | Y |
| Alternative | HEAT | ATMN95 | lag0 and lag1 | 1.351 (1.223, 1.493) | 223 | 5901 | Y | Y |  |  |
|  |  |  | lag1 and lag2 and lag3 |  |  |  |  | Y | Y | Y |
| Alternative | HEAT | ATMN95 | lag0 and lag1 and lag2 and lag3 | 1.238 (1.092, 1.402) | 92 | 5849 | Y | Y | Y | Y |
| Logic regression | STROKE | TMX98 | lag0 and lag1 and  (not lag2) and lag3 | 1.257 (1.061, 1.477) | 5 | 16747 | Y | Y | N | Y |
| Alternative | STROKE | TMX98 | lag0 and lag1 and  lag2 and lag3 | 1.067 (0.950, 1.195) | 21 | 16720 | Y | Y | Y | Y |
| Logic regression | RENAL | ATMN95 | lag0 and lag3 | 1.025 (1.011, 1.039) | 255 | 18888 | Y |  |  | Y |
|  |  |  | (not lag2) and lag3 |  |  |  |  |  | N | Y |
| Alternative | RENAL | ATMN95 | lag0 and lag1 and lag2 and lag3 | 1.037 (1.015, 1.058) | 92 | 18879 | Y | Y | Y | Y |

**Table S3.** Summary of extreme heat metrics from truncated continuous versus continuous temperature metric and their short-term associations with warm-season emergency department visits in Atlanta, 1993 to 2012. Relative risk estimates and 95% confidence intervals (CI) were from time-series models adjusting for either truncated or non-truncated continuous temperature.

|  | Truncated Heat Metric | | | | | Non-Truncated Heat Metric | | | | |
| --- | --- | --- | --- | --- | --- | --- | --- | --- | --- | --- |
| Disease | Metric and Threshold | Extreme Heat Metric | Relative risk  (95% CI) | Frequency  (days) | Q-AIC | Metric and Threshold | Continuous Extreme Heat Metric | Relative risk  (95% CI) | Frequency  (days) | Q-AIC |
| HEAT | ATMN95 | (lag1 and (lag0 or lag3)) | **1.400 (1.269, 1.544)** | 237 | 5919 | TMN99 | lag1 | 1.061 (0.926, 1.213) | 63 | 6178 |
| STK | TMX98 | ((lag1 and lag3) and ((not lag2) and lag0)) | **1.257 (1.061, 1.477)** | 5 | 16748 | TMX98 | (((not lag2) and lag3) and lag1) | **1.176 (1.024, 1.344)** | 7 | 16747 |
| ARF | ATMN95 | (lag0 and lag1) | **1.052 (1.021, 1.085)** | 190 | 14481 | TMX95 | (lag1 and (not lag3)) | 1.024 (0.998, 1.051) | 174 | 14497 |
| RENAL | ATMN95 | (lag3 and ((not lag2) or lag0)) | **1.025 (1.011, 1.039)** | 255 | 18882 | ATMN98 | lag1 | 1.007 (0.988, 1.027) | 146 | 18892 |
| FLEL | ATMN95 | lag1 | **1.030 (1.016, 1.045)** | 335 | 17792 | ATMN95 | lag1 | **1.017 (1.002, 1.032)** | 335 | 17929 |
| CIRC | ATMX95 | lag1 | 0.996 (0.987, 1.004) | 363 | 18214 | ATMX95 | lag1 | 0.996 (0.988, 1.005) | 363 | 18210 |
| CHF | ATMN98 | lag0 | 0.976 (0.947, 1.007) | 146 | 18521 | ATMN98 | lag0 | 0.978 (0.945, 1.013) | 146 | 18514 |
| IHD | TMN99 | lag0 | 1.013 (0.986, 1.041) | 63 | 19771 | ATMN99 | (lag2 and ((lag1 and lag3) and (not lag0))) | **1.089 (1.005, 1.179)** | 6 | 19806 |
| MI | TMN99 | lag0 | 1.044 (0.964, 1.131) | 63 | 15331 | TMN98 | lag1 | **1.074 (1.009, 1.144)** | 130 | 15344 |
| DIA | TMN95 | lag2 | 1.009 (0.996, 1.021) | 238 | 19967 | TMN99 | (not lag0) and lag1 | **1.025 (1.001, 1.050)** | 43 | 19997 |
| HT | ATMX95 | (((not lag3) and lag1) and (lag0 or lag2)) | **0.988 (0.977, 0.999)** | 128 | 17402 | ATMN98 | lag1 | 0.997 (0.985, 1.009) | 146 | 17346 |
| INTERN | ATMN95 | lag3 | **1.008 (1.002, 1.015)** | 335 | 9854 | ATMN99 | lag3 | 0.990 (0.979, 1.001) | 74 | 9864 |
